# Cardiac and hepatic role of r-AtHSP70: basal effects and protection against ischemic and sepsis conditions

**DOI:** 10.1111/jcmm.12491

**Published:** 2015-04-23

**Authors:** Teresa Pasqua, Elisabetta Filice, Rosa Mazza, Anna Maria Quintieri, Maria Carmela Cerra, Rina Iannacone, Donato Melfi, Cesare Indiveri, Alfonsina Gattuso, Tommaso Angelone

**Affiliations:** a Department of Biology, Ecology and Earth Sciences, University of CalabriaArcavacata di Rende (CS), Italy; b National Institute of Cardiovascular ResearchBologna, Italy; c ALSIA-Research Center Metapontum AgrobiosMetaponto (MT), Italy

**Keywords:** heat shock protein, *Arabidopsis thaliana*, heart, liver, cardioprotection, sepsis

## Abstract

Heat shock proteins (HSPs), highly conserved in all organisms, act as molecular chaperones activated by several stresses. The HSP70 class of stress-induced proteins is the most studied subtype in cardiovascular and inflammatory disease. Because of the high similarity between plant and mammalian HSP70, the aim of this work was to evaluate whether recombinant HSP70 of plant origin (r-AtHSP70) was able to protect rat cardiac and hepatic function under ischemic and sepsis conditions. We demonstrated for the first time that, in *ex vivo* isolated and perfused rat heart, exogenous r-AtHSP70 exerted direct negative inotropic and lusitropic effects *via* Akt/endothelial nitric oxide synthase pathway, induced post-conditioning cardioprotection *via* Reperfusion Injury Salvage Kinase and Survivor Activating Factor Enhancement pathways, and did not cause hepatic damage. *In vivo* administration of r-AtHSP70 protected both heart and liver against lipopolysaccharide-dependent sepsis, as revealed by the reduced plasma levels of interleukin-1β, tumour necrosis factor alpha, aspartate aminotransferase and alanine aminotransferase. These results suggest exogenous r-AtHSP70 as a molecular modulator able to protect myocardial function and to prevent cardiac and liver dysfunctions during inflammatory conditions.

## Introduction

Heat shock proteins (HSPs) represent a family of highly conserved molecular chaperones, classified according to their molecular sizes, whose main function consists in preventing misfolding and aggregation of nascent polypeptides or facilitating protein folding in both eukaryotic and prokaryotic organisms 1,2. They also protect proteins during exposure to stressful situations such as heat shock, which causes proteins unfolding 3,4. Recently, a revised nomenclature for human HSP families was proposed which includes the following families HSP110, HSP90, HSP70, HSP60 (chaperonins), HSP40 and Small HSPs 5.

The HSP70 family contains multiple members which generally have overlapping function in the cell, albeit some members can provide tissue-specific expression and function 6,7. Their best described functions are to mediate the correct protein folding, thus maintaining protein homeostasis, and to enhance cell survival following a multitude of stresses 8–15. The HSP70 class is also the most studied stress-induced proteins in cardiovascular and inflammatory disease 16. HSP70 induction provides cardioprotection after ischemia-reperfusion injury even if the exact mechanisms are unknown. A correlation between elevated HSP70 levels, induced by a short period of ischemia, and improved myocardial function after subsequent ischemia has been reported both *in vivo* and *in vitro* 17. In particular, preconditioning of hearts by hyperthermia or ischemia up-regulates the intracellular expression of HSP70 that seems to be defensive for the myocardium enhancing cellular tolerance to further stress 18. This action may be ascribed to protective effects of HSP70 on mitochondrial function also preventing apoptotic cell death induced by simulated ischemia-reperfusion 19. Likewise, inducible HSP70 is a key component of endogenous pathways that limit the extent of myocardial damage in ischemia–reperfusion injury after cardiac surgery 20,21 and it is also involved in cardiac growth and hypertrophy 18. HSP70 may exert a protective mechanism not only in acute damaging situations, *e.g*. associated with ischemia and reperfusion following coronary bypass grafting, but also in patients with chronic ‘stress’, as in chronic heart failure 22.

HSP70 also shows immunoregulatory properties with both anti- and pro-inflammatory effects. While intracellular HSP70 is able to counteract pro-inflammatory signalling cascades and inflammation through NFκB inhibition, its extracellular action is mainly pro-inflammatory 16. Previous studies have reported that exogenous mammalian HSP70 (both isolated from bovine muscle and pure recombinant human HSP70) decreases lipopolysaccharide (LPS)-induced sepsis manifestations playing an important role in innate immunity modulation and stimulation of endogenous protective mechanisms, both at the cellular and organism levels 23,24. Also the hepatic response to stress may involve the synthesis and accumulation of HSP70. Its induction seems to have beneficial effects in conditions such as ischemia-reperfusion injury in human liver 25, physical stress in rat liver 26 and in LPS-mediated inflammatory response 27.

The crystal structure of HSP70 from different organisms including *Escherichia coli* (DnaK) 28, *Caenorhabditis elegans* 29, and mammals 30, has been now resolved revealing a very high degree of homology in the tertiary structure. Recently, it has been demonstrated that plant HSP70 is structurally and functionally similar to its mammalian homologue 31.

In this study an inducible HSP70 cDNA was isolated from *Arabidopsis thaliana* (AtHSP70) and produced in *E. coli*. This may represent the first step to evaluate the activity and influence of a plant protein on mammalian heart and liver. Plants offer many advantages respect to mammalian cultures such as low costs, possibility to increase the amount of desired molecule and absence of human pathogens. The ‘plant system’ was demonstrated to be effective for the production of several vaccines, antibodies and therapeutic proteins (for review see 32). Moreover, differently from exhausted microorganism, plant waste could be recycled as, for example, biofuels. The final goal will be the production of the plant protein in a plant system. In fact, in the last decade the use of plants as bioreactors has greatly advanced and now pharmaceuticals obtained from plant cells are already in the market 33.

Here, we examined whether recombinant AtHSP70 (r-AtHSP70) purified from bacterial cultures was able to exert: (*i*) cardiac and hepatic effects at basal conditions; (*ii*) myocardial protection against ischemia/reperfusion (I/R) injury and its mechanism of action and (*iii*) cardiac and hepatic protection against LPS-dependent sepsis. Both liver and heart represent simultaneous targets of a number of systemic conditions including infection and inflammation that often lead to adverse outcomes 34. For this reason, they represent good tools for investigating the systemic role of r-AtHSP70 during sepsi.

## Materials and methods

### Animals

Male Wistar rats (Harlan Laboratories, Udine, Italy), weighing 180–250 g were used. Animal care, sacrifice and experiments were done in accordance with the U.S. National Institutes of Health Guide for the Care and Use of Laboratory Animals (NIH Publication No. 85-23, revised 1996) and are in accordance with the Italian law (DL 116, January 27, 1992). The scientific project was supervised and approved by the local ethical committee.

### HSP70 Cloning and purification

#### cDNA isolation

The coding region of the gene HSP70-4 (NP_187864) was amplified by PCR from cDNA obtained from *Arabidopsis thaliana* tissues submitted to heat stress (40°C for 30 min.) using the following primers: AtHSP70F: 5′-TAATGGCGGGTAAGGTGAA-3′; AtHSP70R: 5′-GCCAAAAGGCTTAATCAACTTC-3′. The cDNA was cloned in the *E. coli* expression vector pET27, resulting in pET27AtHSP70, and inserted in BL21 cells by chemical transformation.

#### Protein extraction and purification

O/N *E. coli* cultures, harvested from 2 l culture, were pelleted by centrifugation at 8000 × g for 15 min., resuspended in sonication buffer (50 mM Tris-Cl, 500 mM NaCl 20 mM imidazole) and disrupted by sonication (90 sec. for 6 times at 60% intensity) using a Vibra Cell sonicator (Sonics). Recombinant AtHSP70 protein was purified by affinity chromatography using the AKTA FPLC system (GE Healthcare, Tampa, FL, USA). In details, total proteins were loaded onto a His-Prep FF16/10 (GE Healthcare) column and washed with elution buffer with increased imidazole concentration (0–500 mM). r-AtHSP70 was eluted at 250 mM imidazole concentration. The recombinant protein was then dialyzed against TrisCl 50 mM pH 7.5 buffer and lyophilized (FreeZone 18 Liter Console Freeze Dry System; LabConco, Kansas City, MO, USA). The purity of r-AtHSP70 was checked by SDS-PAGE and Western blot.

### Isolated heart preparation

The hearts were isolated and perfused as previously described 35. Rats were anaesthetized by intraperitoneal injection (i.p.) of ethyl carbamate (2 g/kg rat). Hearts were rapidly excised and immediately transferred in ice-cold buffered Krebs–Henseleit solution (KHs) for immediate cannulation of the aorta through a glass cannula. Retrograde perfusion was conducted at constant flow-rate (12 ml/min.). To avoid fluid accumulation, the apex of the left ventricle (LV) was pierced. A water-filled latex balloon, connected to a pressure transducer (BLPR; WRI, Inc., Sarasota, FL, USA), was inserted through the mitral valve into the LV, to allow the recording of cardiac mechanical parameters. A second pressure transducer located above the aorta was used to record coronary pressure (CP). The perfusion solution consisted of a modified non-recirculating KHs containing (in millimoles) NaCl 113, KCl 4.7, NaHCO_3_ 25, MgSO_4_ 1.2, CaCl_2_ 1.8, KH_2_PO_4_ 1.2, glucose 11, mannitol 1.1, Na-pyruvate 5 (pH 7.4; 37°C; 95% O_2_/5% CO_2_). Hemodynamic parameters were assessed using a PowerLab data acquisition system and analysed using a Chart software (ADInstruments, Oxford, UK).

### *Ex vivo* experiments

#### Basal conditions

As previously detailed 35, inotropism was evaluated in terms of the LV pressure (LVP; mmHg, index of contractile activity) and the maximal value of the first LVP derivative [+(LVdP/dT)max; in mmHg/sec., index of the maximal rate of LV contraction]. The lusitropism state was assessed on the basis of the maximal rate of LVP decline [−(LVdP/dT)max; mmHg/sec.] and the T/−T ratio [obtained by +(LVdP/dt)max/−(LVdP/dt)max]. Coronary vasomotility was evaluated in terms of CP (mmHg), while heart rate (HR) changes (beats/min) were used to estimate chronotropism.

#### r-AtHSP70-stimulated preparations

After 20 min. of equilibration, dose–response curves were generated by perfusing cardiac preparations with KHs supplemented with increasing concentrations of r-AtHSP70 (10^−12^–10^−8^ M), each of them for 10 min. Repetitive exposure of each heart to a single concentration (10^−10^ M) of r-AtHSP70 revealed absence of desensitization (data not shown).

#### Ischemia/reperfusion protocols

Each heart was allowed to stabilize for 20 min.; at this time, baseline parameters were recorded. After stabilization, hearts were randomly assigned to one of the treatment groups described below and then subjected to 30-min. of global, no-flow ischemia followed by 120-min. of reperfusion (I/R). Pacing was discontinued at the beginning of the ischemic period and restarted after the third minute of reperfusion 36–38.

##### Cardiac function and infarct size studies

1. In the first group (I/R group, *n* = 6), hearts were stabilized and subjected to I/R protocol only.
2. In the second group (PostC group, *n* = 6), hearts underwent to a postconditioning protocol (*i.e*. 5 cycles of 10-sec. reperfusion and ischemia at beginning of reperfusion).
3. In the third group (r-AtHSP70 group, *n* = 6), r-AtHSP70 (10^−10^ M) was infused for 20-min. at the beginning of 120-min. reperfusion.
4. In the fourth group (r-AtHSP70 plus inhibitors, *n* = 6 for each group), hearts were perfused with r-AtHSP70 plus one of the following inhibitors: Wortmannin (WT, 10^−7^ M), a potent phosphatidylinositol 3-kinase (PI3K) inhibitor; PD98059 (PD, 10^−8^ M), a specific inhibitor of ERK1/2; 5-hydroxydecanoate (5HD, 10^−5^ M), a mitoKATP channels blocker. Perfusion with inhibitors started 5-min. before ischemia and continued during the early 20-min. of reperfusion in the presence of r-AtHSP70 (10^−10^ M). A set of experiments was performed with inhibitors alone used as control. The concentration of r-AtHSP70 was chosen on the basis of a preliminary dose–response curve as the dose that induced the highest infarct size reduction (data not shown).

Cardiac performance before and after ischemia was evaluated for inotropism by analysing, as index of contractile activity, the recovery of LVP (mmHg), and for contracture by analysing the left ventricle end-diastolic pressure (LVEDP, mmHg). Contracture can be defined as an increase in LVEDP of 4 mmHg above the baseline level 36–38.

##### Assessment of myocardial injury

To obtain infarct size, hearts were rapidly removed from the perfusion apparatus at the end of reperfusion, and the left ventricle was dissected into 2- to 3-mm circumferential slices. After 20-min. of incubation at 37°C in 0.1% solution of nitro blue tetrazolium in phosphate buffer, unstained necrotic tissue was carefully separated from stained viable tissue by an independent observer who was not aware of the nature of the intervention. The weights of the necrotic and non-necrotic tissues were then determined, and the necrotic mass was expressed as a percentage of total LV mass, including septum 37.

##### Lactate dehydrogenase

Since in isolated rat hearts ischemic postconditioning is known to reduce the production of lactate dehydrogenase (LDH) during reperfusion 37, the release of this enzyme during each experimental group was tested. Samples of coronary effluent were withdrawn with a catheter inserted into the right ventricle *via* the pulmonary artery. Samples were taken during reperfusion. LDH release was measured as described by Penna *et al*. 37. Data are expressed as cumulative values for the entire reperfusion period. LDH was evaluated by an ELISA system using rat kits (Blue Gene, Shanghai, China).

### Western blotting analysis

To evaluate differences in protein phosphorylation or expression, cardiac ventricles, obtained after perfusion (10 min.) with KHs alone or with a single concentration of r-AtHSP70 (10^−10^ M) or belonging to the three I/R groups, were used (120 min.). Samples were homogenized in ice-cold RIPA buffer (Sigma-Aldrich, Milan, Italy) containing a mixture of protease inhibitors (1 mmol/l aprotinin, 20 mmol/l phenylmethylsulfonyl fluoride and 200 mmol/l sodium orthovanadate). Then homogenates were centrifuged at 200 × g for 10 min. at 4°C for debris removal. Protein concentration was determined using Bradford reagent according to the manufacturer’s recommendations (Sigma-Aldrich). Equal amounts of proteins were separated on 8% SDS-PAGE gels [for p-endothelial nitric-oxide synthase (p-eNOS), eNOS, Inducible nitric oxide synthase (iNOS), 60 μg] or on 10% SDS-PAGE gels (for p-Akt, Akt, NFκB p50, GSK3b, p-GSK3b, p-signal transducer and activator of transcription 3 (p-STAT3), STAT3, p-ERK1/2, ERK1/2, 50 μg), transferred to PVDF membranes, blocked with non-fat-dried milk and incubated overnight at 4°C with a polyclonal rabbit anti-p-eNOS antibody (Ser-1177), or a polyclonal goat anti-iNOS antibody, or with a polyclonal rabbit anti-p-Akt antibody (Ser-473), or monoclonal mouse anti-NFκB p50 antibody, or polyclonal goat anti-p-GSK3b antibody (Ser-9), or monoclonal mouse anti-p-ERK1/2 antibody (Tyr 204), or plyclonal goat p-STAT3 antibody (Ser 727; Santa Cruz Biotechnology, Santa Cruz, CA, USA) diluted 1:1000 in TBS-T containing 5% non-fat dry milk. The anti-rabbit and anti-goat peroxidase-linked secondary antibodies (Santa Cruz Biotechnology) were diluted 1:2000 in TBS-T containing 5% non-fat dry milk. Polyclonal rabbit anti-eNOS and anti-Akt, and polyclonal goat anti-β-actin antibodies were used as loading controls (Santa Cruz Biotechnology).

Immunodetection was performed with an enhanced chemiluminescence kit (ECL PLUS, GE Healthcare Europe GmbH, Milan, Italy) Autoradiographs were obtained by exposure to X-ray films (Hyperfilm ECL; GE Healthcare Europe GmbH, Milan, Italy). Immunoblots were digitalized and the densitometric analysis of the bands was carried out using NIH IMAGE 1.6 for a Macintosh computer based on 256 grey values (0 = white; 256 = black).

### Liver isolation and perfusion

A midline incision was made to the abdomen, and portal vein was cannulated and flushed with Krebs–Henseleit buffer in a non-recirculating system as previously described 39.

After flushing had begun, the inferior vena cava was ligated above the right renal vein and cut distally. During flushing, the liver was dissected free from the rat, moved to the perfusion apparatus, positioned in a plexiglass chamber to start a monovascular anterograde perfusion. The total time elapsed between the cessation of the portal blood circulation and the initiation of the perfusion was less than 2 min. The perfusion medium and conditions were the same used for the heart preparation. A pressure transducer (Edwards Lifesciences, Irvine, CA, USA) were placed on line, immediately ahead of the portal inlet cannula, to continuously monitor hepatic portal pressure (PP). The pressure transducer was connected to a PowerLab (4SP) linked to a computer using the Chart version 5.0.1 for Windows software (ADInstruments, Mountain View, CA, USA). The average PP was continuously sampled, recorded, and afterward blindly analysed under code. The perfused rat liver preparation was allowed to stabilize for 20 min. before the studied substances were added.

### *In vivo* experiments

#### LPS treatment

To evaluate the possibility of r-AtHSP70 to counteract *E. coli* LPS (Sigma-Aldrich) -induced sepsis, animals were divided in three groups: (*i*) treatment (i.p.) with a placebo of saline solution; (*ii*) treatment by a single dose (i.p.) of LPS (5 mg/kgbw); (*iii*) treatment with a single dose of LPS plus r-AtHSP70 (5 mg/kgbw). Animals were killed after 48 hrs so to permit plasma collection and heart and liver performance evaluation.

##### Plasma collection

Blood samples were collected from the abdominal aorta with heparinized syringe. Plasma was then separated by centrifugation at 4°C and stored at −80°C until being assayed.

##### Liver perfusate collection

The flow was maintained at a rate of 3 ml/min./g liver. From the total volume of the perfusate, a small amount of perfusate (500 μl) was collected and stored at −80°C until being assayed.

##### Biochemical analysis

Both blood and liver perfusate samples were used to measure the levels of aspartate aminotransferase (AST) and alanine aminotransferase (ALT). ALT and ASP were evaluated by an ELISA system using rat kits (Blue Gene). Tumour necrosis factor alpha (TNF-α) and interleukin (IL)-1β levels in plasma were evaluated by an ELISA system using rat kits (Thermo Scientific, Rockford, IL, USA).

### Statistics

Data are expressed as the mean ± SEM. Since each heart represents its own control, the statistical significance of differences within a group was assessed using the anova test (*P* < 0.05). Comparison between groups was made by using a one-way anova followed by the Bonferroni correction for post-hoc *t*-tests; statistical significance was concluded at *P* < 0.05.

## Results

### Protein purification

**Figure 1:**
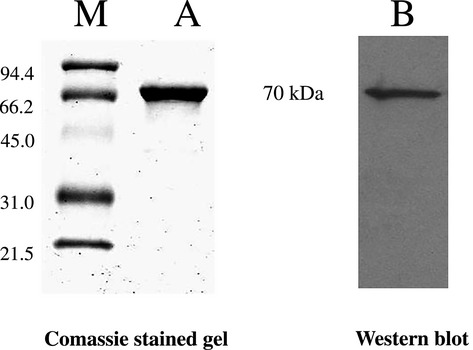
SDS-PAGE and Western blot of purified HSP70 protein from *Escherichia coli* coltures (M = molecular marker). 5 μg of purified protein were loaded on the SDS-PAGE gel and 0.5 μg were loaded on WB.

Transfection of pET27-AtHSP70 in BL21 cells resulted in the accumulation of recombinant protein. SDS-PAGE and Western blot analysis (Fig.1) performed on the purified protein revealed the presence only of the target protein with the expected molecular weight.

### *Ex vivo* experiments

#### Basal conditions

Cardiac parameters, obtained after 20 min. equilibration, are in Table1. The endurance and stability of the preparations, analysed by measuring the performance variables every 10 min., showed that each heart was stable up to 180 min.

**Table 1:** Basal cardiac parameters

LVP (mmHg)	HR (beats per min)	EDVP (mmHg)	RPP (mmHg beats per min)	+(LVdP/dt)_max_ (mmHg/sec.)	−(LVdP/dt)_max_ (mmHg/sec.)	Time to peak (sec.)	HTR (sec.)	T/−t (mmHg/sec.)	CP (mmHg)	Pressure perfusion (mmHg)
89 ± 3	280 ± 7	5–8	2.5 ± 0.1 10^4^	2492 ± 129	−1663 ± 70	0.08 ± 0.01	0.05 ± 0.01	1.498 ± 1.84	63 ± 3	100

For abbreviation see Materials and methods.

#### Dose–response curves of r-AtHSP70

**Figure 2:**
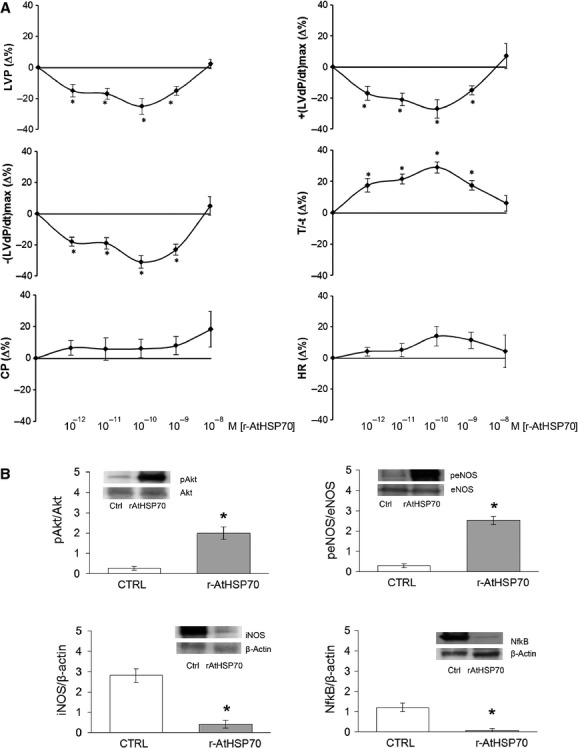
(A) Dose–response curves of r-AtHSP70 (10^−12^–10^−8^ M) on LVP, +(LVdP/dT)max, −(LVdP/dT)max, T/−T, CP and HR, on Langendorff perfused rat heart preparations. Percentage changes were evaluated as means ± SEM of seven experiments. Significance of difference from control values was evaluated by one-way anova; *P < 0.05. (B) Immunoblots showing Akt and eNOS phosphorylation, and iNOS and NFκB expression in cardiac ventricles (*n* = 4). Significance of difference from control values was evaluated by one-way anova; *P < 0.05.

To evaluate whether the exogenous r-AtHSP70 affects basal cardiac parameters, heart preparations were exposed to increasing concentrations (from 10^−12^ to 10^−8^ M) of the protein after 20 min. of stabilization. Since the protein-induced effects remained stable for 15–20 min., cardiac parameters were measured after 10 min. of exposure to each different concentration. r-AtHSP70 induced negative inotropic and lusitropic effects from 10^−12^ to 10^−9^ M [indicated by significative reduction of LVP, +(LVdP/dt)max, −(LVdP/dt)max and increase of T/−t] that disappeared at the highest dose tested (10^−8^ M), describing U-shaped curves (Fig.2A). The concentration-response curves show that neither CP nor the HR were modified by protein (Fig.2A).

#### Western blot

The immunoblotting analysis of r-AtHSP70 (10^−10^ M) treated hearts, revealed changes in protein phosphorylation or expression respect to the untreated hearts. Data showed an increase in Akt and eNOS phosphorylation and a reduction in iNOS and NFκB (Fig.2B).

#### Improvement of post-ischemic cardiac function by r-AtHSP70

**Figure 3:**
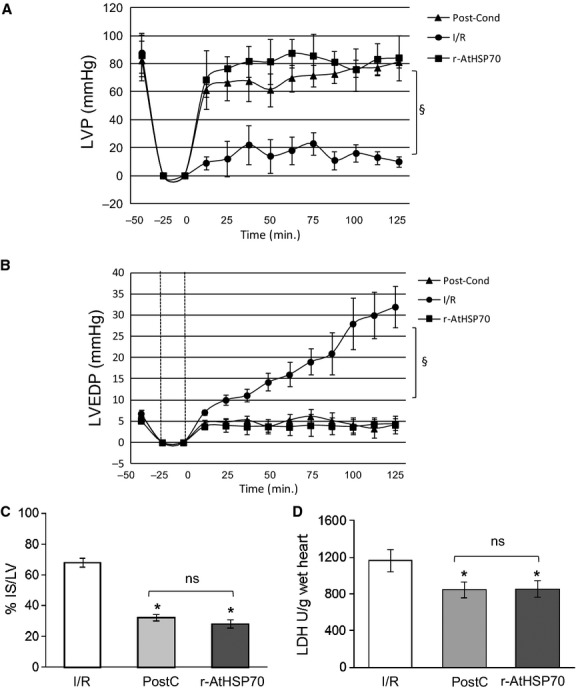
LVP (A) and LVEDP (B) variations. Data are expressed as changes of LVP and LVEDP values (mmHg) from the stabilization to the end of the 120 min. of reperfusion with respect to the baseline values for each group. Vertical lines indicate ischemic administration. Comparison between groups, ^§^*P* < 0.05. (C) Infarct size. The amount of necrotic tissue measured after 30-min. global ischemia and 120-min. reperfusion is expressed as percentage of left ventricle (IS/LV %). (D) LDH release in I/R, PostC and r-AtHSP70 PostC. Significance of difference was evaluated by one-way anova; *P < 0.05 with respect to I/R. *N* = 6 for each group.

The possibility that plant HSP70 elicits cardioprotection was investigated by comparing the effects induced by PostC manoeuvres with those elicited by the peptide administered after I/R. Both systolic and diastolic functions were analysed. Systolic function is represented by the level of inotropic activity (*i.e*. LVP recovery). Hearts of the I/R group presented a limited LVP recovery; in fact, at the end of reperfusion, LVP was 16.5 ± 3.8 mmHg instead of baseline value, 87.75 ± 13.9 mmHg. At the end of PostC protocols, LVP was 70 ± 5 mmHg instead of baseline value, 82 ± 5 mmHg (Fig.3A). r-AtHSP70 (10^−10^ M) markedly improved LVP recovery during reperfusion, being LVP at the end of reperfusion 78 ± 16 mmHg (baseline value 85 ± 14 mmHg; Fig.3B). Diastolic function is represented by the level of contracture (*i.e*. LVEDP 4 mmHg or more above baseline level) 40 I/R markedly increased LVEDP (from 6.8 ± 0.9 mmHg in the baseline to 32 ± 10 mmHg at the end of reperfusion; Fig.3B). LVEDP was not significantly modified by PostC, being 8.8 ± 2 mmHg at the end of the experimental protocol (Fig.3B). During reperfusion, r-AtHSP70 abolished contracture development; in fact, LVEDP at the end of reperfusion was 2.54 ± 1.42 mmHg (Fig.3B).

Total infarct size was expressed as a percentage of LV mass (Fig.3C). LV mass was similar in all groups (LV weight was 1027 ± 24 mg). Infarct size was 68 ± 3% in I/R, 32 ± 2% in PostC.

To confirm infarct size data, LDH release was analysed in three hearts for the I/R group or for the r-AtHSP70 group. The results showed that in hearts of I/R group the cumulative LDH release during reperfusion was 1251 ± 115 U/g wet heart, while in r-AtHSP70 group the release of LDH was 923 ± 79 U/g wet heart, significantly lower respect to the I/R group (Fig.3D).

#### Involvement of RISK/SAFE pathways and mitoKATP channels in r-AtHSP70-induced cardioprotection

**Figure 4:**
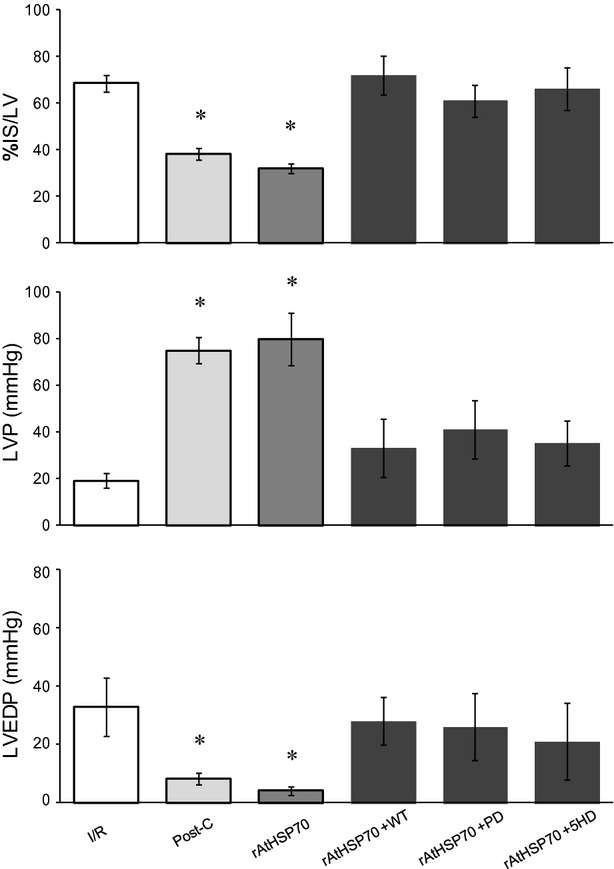
Percent variation in Infarct Size(IS)/LV and absolute values of systolic and diastolic function (LVP and EDP) at the end of 120-min. reperfusion of following groups: I/R, PostC, r-AtHSP70 alone and r-AtHSP70 plus inhibitors (WT, PD and 5HD). Significance of difference was evaluated by one-way anova; *P < 0.05 with respect to I/R. *N* = 6 for each group.

To verify the involvement of prosurvival kinases of Reperfusion Injury Salvage Kinase (RISK) pathway and mitoKATP channels, r-AtHSP70 –dependent improvement of postischemic LVP, EDP and infarct size was analysed in the presence of specific inhibitors. When the hearts were co-treated with r-AtHSP70 plus the inhibitor of PI3K or ERK1/2 (WT or PD), or with the blocker (5HD) of mitoKATP channels, cardioprotection on systolic and diastolic function and infarct size was abolished (Fig.4). In hearts perfused with inhibitors only, LVP, EDP and infarct size were not different from I/R group (data not shown).

**Figure 5:**
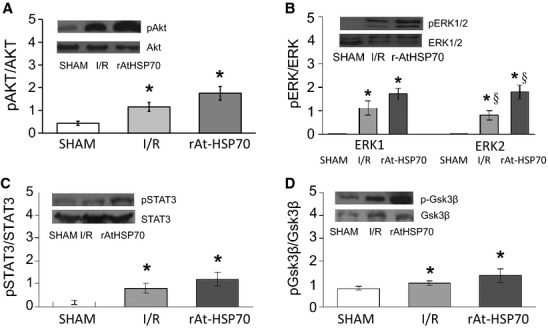
Western blot analysis for RISK and SAFE pathway. Representative Western blots and relative densitometry showing that HSP70 in early reperfusion induces an increase in the phosphorylation of Akt (A), ERK1/2 (B), STAT3 (C), and GSK3β (D) with respect to I/R group. Total kinases and phosphokinases for each heart are normalized to the total protein levels. Values are mean ± SEM. *P < 0.05 with respect to control conditions; ^§^*P* < 0.05 (Post-HSP70 *versus* I/R), *N* = 4 for each group.

RISK and Survivor Activating Factor Enhancement (SAFE) pathways were assessed by Western blot analysis. Representative bands and densitometric analysis of the scanned blots were detected after 20 min. of reperfusion. Phosphorylation data were normalized with respect to the total protein. The infusion of r-AtHSP70 in PostC enhanced phosphorylation for all the proteins analysed. In particular, the infusion with HSP70 after the global ischemia induced an evident activation/phosphorilation of Akt (A), Erk1/2 (B), STAT3 (C) and Glycogen synthase kinase 3 beta (GSk3β) (D) with respect to I/R group (Fig.5).

#### Hepatic effects

**Figure 6:**
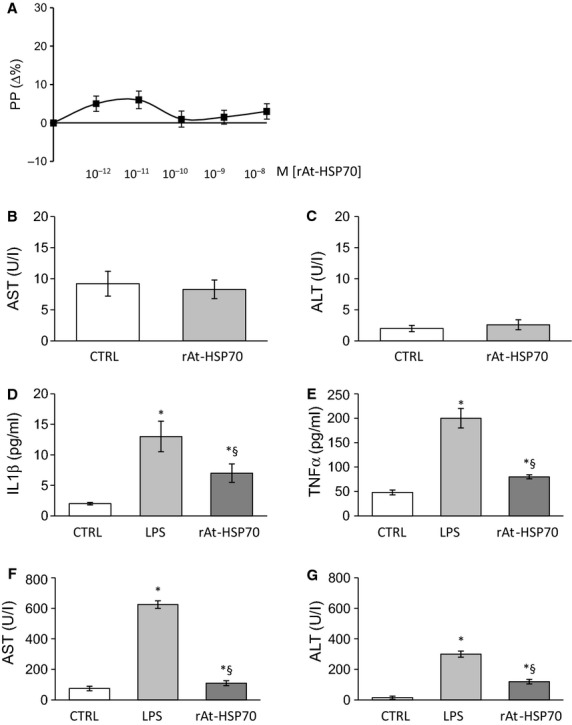
(A) Dose–response curves of r-AtHSP70 (10^−12^–10^−8^ M) on portal pressure (PP) of the isolated and perfused rat liver. ELISA analysis of AST (B) and ALT (C) in liver collected perfusate. Biochemical analysis of plasma levels regarding IL-1β (D), TNF-α (E), AST (F) and ALT (G) after chronic treatment with placebo, LPS and LPS plus HSP70. *P < 0.05 (LPS or LPS plus HSP70 *versus* control); ^§^*P* < 0.05 (LPS plus HSP70 *versus* LPS alone), *N* = 6.

Dose–response curves of r-AtHSP70 (10^−12^–10^−8^ M) performed on the isolated and perfused liver showed that the protein was not able to modify significantly the PP (Fig.6A). Analysis of the perfusion buffer, collected after each experimental protocol, indicated that neither AST (Fig.6B) nor ALT (Fig.6C) release was modified by r-AtHSP70 treatment respect to the untreated organ.

### *In vivo* experiments

#### Plasma analysis

Analysis performed on the plasma collected from animals subjected to treatment with placebo, or LPS (5 mg/kgbw) or LPS plus r-AtHSP70 (5 mg/kgbw), showed a significant increase in plasma levels of IL-1β (Fig.6D), TNF-α (Fig.6E), AST (Fig.6F), and ALT (Fig.6G) in LPS-treated animals respect to the placebo, while the contemporary administration of r-AtHSP70 was able to counteract these augmentations. A control group with r-AtHSP70 alone did not induce significant changes of above plasma determinations (data not shown).

#### Performance evaluation

**Figure 7:**
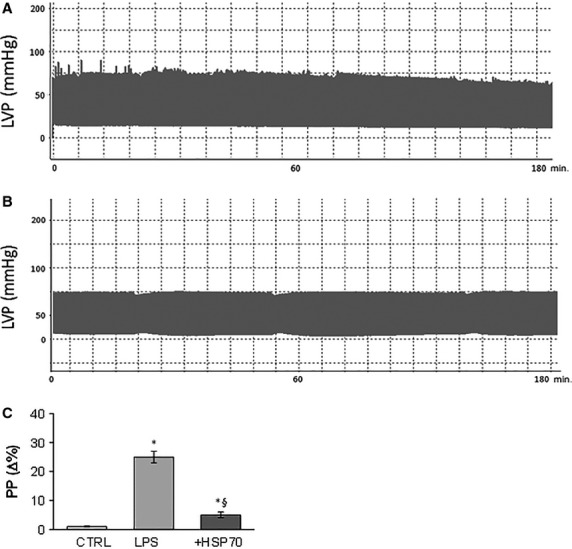
Cardiac systolic performance after (A) LPS (5 mg/kgbw) and (B) LPS plus HSP70 (5 mg/kgbw) i.p. treatment. Evaluation of portal pressure (PP) in the isolated liver after placebo, LPS and LPS plus HSP70 i.p. treatment (C). *P < 0.05 (LPS or LPS plus HSP70 *versus* control); ^§^*P* < 0.05 (LPS plus HSP70 *versus* LPS alone), *N* = 6.

Evaluation of cardiac performance showed a reduction in the systolic function and in the endurance of the cardiac preparation after i.p. treatment with LPS; on the other hand, administration of LPS plus r-AtHSP70 showed a stable and well-functioning heart (Fig.7A and B). Regarding the hepatic effects, LPS treatment alone increased PP in the isolated liver while LPS plus r-AtHSP70 counteracted this rise (Fig.7C). In the rats treated with r-AtHSP70 alone no changes were observed on both heart and liver performance (data not shown).

## Discussion

This study indicates that r-AtHSP70 (*i*) directly modulates basal heart performance, (*ii*) protects against I/R myocardial injuries by RISK and SAFE pathways and (*iii*) counteracts LPS-dependent inflammatory pathways in both heart and liver.

The recombinant plant protein extracted from *E. coli* (r-AtHSP70) and homogeneously purified was produced by the ‘Metapontum Agrobios’ research center which has developed tools for the extraction of large quantities of plant HSP70 from bio-mass (mainly leaves) enriched with the inducible forms of HSP70 by using physical methods (heat stress) or by the expression of said genes in heterologous systems (bacteria, yeast, plants) using genetic engineering in the prospective of using plant proteins for pharmaceutical applications.

### r-AtHSP70 *ex vivo* effects

To the best of our knowledge this study represents the first evidence that, under basal conditions, exposure to exogenous r-AtHSP70 affects contractility and relaxation in a dose-dependent manner. In particular, the negative inotropic effect, obtained without changing the HR or the CP, is consistent with a direct inotropic effect of the protein on the myocardium independent from coronary reactivity. These actions disappear at higher concentrations generating an U-shaped curve. This phenomenon may suggest the activation of counter- regulatory mechanisms activated at high concentrations which abolish the r-AtHSP70 effects. The mechanism underlying this behaviour is unknown. However, other biological agents such as endostatin 41, interferon 42, CGA 40 and its biphasic influence on blood pressure levels and catecholamine secretion in mice 43 exhibit U-shaped concentration/response curves. Further work is necessary to address this issue.

These cardiac effects were mediated by Akt/eNOS pathway. It is known that endogenous HSP70, together with HSP90, are largely expressed in myocardial cells and are important for cardiac response to stress. HSP70 improves functional recovery and reduces infarct size after ischemia through the protection of mitochondrial function, while HSP90 exerts pro-angiogenic effects through Akt and eNOS 44 increasing eNOS activity and nitric oxide release 45, by a calmodulin dependent mechanism 46. It was also reported a HSP90-independent increase in basal eNOS enzymatic activity that promotes interaction between HSP70 and eNOS 47. The effects observed on cardiac preparations by r-AtHSP70 may be attributable to the same mechanism of the endogenous protein. Indeed, under basal conditions, r-AtHSP70 treatment significantly augmented p-Akt/p-eNOS expression thus supporting eNOS activation.

Intracellular HSP70 plays cytoprotective and anti-inflammatory functions by avoiding NFκB activation, which has severe implications for immunity, inflammation, cell survival and apoptosis 48,49. HSP70 confers cardiovascular protection during endotoxemia *via* inhibition of iNOS expression by preventing NFκB activation 50. Prolonged activation of NFκB is involved in the systolic and diastolic dysfunction by enhancing inflammatory cytokines such as TNF-α, IL-1 and interleukin-6 51. Protective effects of extracellular mammalian HSP70 have been demonstrated in experiments such as LPS-treated rats 23, or in the septic shock model suppressing neutrophils and macrophages activation 24. Of note, in our experiments treatment with r-AtHSP70 reduced the levels of both NFκB and iNOS confirming an anti-inflammatory role of the exogenous protein under basal conditions. However, the mechanism of action remains to be defined, *i.e*. if it interacts with membrane receptors or it crosses the cell membrane exerting its effects on intracellular targets.

### Cardioprotection

Ischemic post-conditioning (IPost), during which repeated brief episodes of I/R are applied at the onset of reperfusion, has emerged as a promising cardioprotective therapy against lethal reperfusion injury 52. By using a pharmacological post-conditioning protocol we observed that r-AtHSP70 given in the early reperfusion, induced a significant protective effect against myocardial I/R injury reducing the infarct size and LDH release. A marked improvement of post-ischemic contractile function and a decrease of contracture development have been also observed.

IPost exerts its cardioprotective effect by activating intrinsic pro-survival signalling cascades such as RISK pathway and the recently described SAFE pathway. The RISK protective mechanisms activated in reperfusion include at least two parallel pathways (ERK1/2 and PI3K-Akt), and converge during reperfusion on GSK3b phosphorylation/inactivation with the involvement of mitoKATP channel opening 53. Our findings suggest that the r-AtHSP70-induced early protection involves this mechanism. In fact, r-AtHSP70 protection was abrogated by blocking each of these elements. The activation of the SAFE pathway includes the activation of the transcription factor STAT-3 54. Very interestingly, r-AtHSP70 significantly increased Akt, Erk1/2, GSk3β, and STAT3 phosphorylation indicative of both RISK and SAFE cascades activation. This suggests r-AtHSP70 as a promising post-conditioning cardioprotective agent attenuating I/R injury. Other post-conditioning agents, such as GLP2 or Catestatin, protect the heart through SAFE and RISK pathways 53,55. Although we have not tested interaction between r-AtHSP70 and other cardioprotective agents, we can speculate that their effects may be additive or exclusive converging on mitochondria as final common target.

### LPS-dependent endotoxic shock

Lipopolysaccharide is a pathogen-associated large molecule responsible for sepsis-related endotoxic shock; heart and liver are adversely affected by LPS and their failure is associated with adverse outcome 34,56. Previous studies in rats showed that administration of either animal 23, or recombinant human HSP70 decreased LPS-induced sepsi 24. PES (HSP70 substrate binding activity inhibitor) prevented LPS-induced increase in serum ALT and AST, reduced iNOS, TNF-α, and IL-6 content in LPS-stimulated mice 27 and rat 57 and favoured human liver recovery from I/R by preventing LPS-induced NFκB activation 25.

r-AtHSP70 counteracted the LPS-induced increases of ALT, AST, IL-1β, and TNF-α plasma levels without affecting *per se* neither enzymes nor inflammatory cytokines, also in agreement with the involvement of HSP70 in LPS-mediated inflammatory responses in the liver.

*Ex vivo* evaluation of cardiac performance after LPS treatment showed that the LPS-dependent reduction in systolic function and endurance disappeared in the presence of co-administration of r-AtHSP70, suggesting a cardioprotective role against endotoxic shock. Yao *et al*. 58 have reported that an increase of HSP70 partly mediates LPS-induced cardioprotection through inhibition of NFκB, and that LPS pretreatment (1.0 mg/kg) could limit myocardial I/R injury. Moreover, LPS (2.5 mg/kg) can protect the heart as late preconditioning (late PC) agent through iNOS activation 59. iNOS-dependent activation by LPS, before lethal ischemia, increases nitric oxide release that may activate the downstream signalling pathway giving cardioprotection. Late PC is a phenomenon which triggers a complex cascade of signalling events including the activation of several cardioprotective factors such as NFκB, iNOS, and cyclooxygenase-2 60. In literature, both low (58: 1 mg/kg; 23,24: 2 mg/kg] and high 61–63: 10–25 mg/kg) doses of LPS are able to induce septic shock. The dose tested in our experiments (5 mg/kg) was able to induce cardiac and liver damage that disappears in presence of r-AtHSP70. Similar doses have been also used by other authors 64.

Since portal hypertension is pathogenically related to liver injury and associated with the activation of inflammatory pathways 65, we have also examined the hepatic pressure after LPS alone and in presence of r-AtHSP70. In contrast to LPS treatment, exogenous r-AtHSP70 *per se* did not modify the portal hepatic pressure (PP), but the co-administration of LPS plus HSP70 abolished the PP increase induced by LPS.

In conclusion, in this study we demonstrated for the first time that exogenous r-AtHSP70 modulates cardiac performance by nitric oxide signalling and induces post-conditioning cardioprotection *via* RISK and SAFE pathways. In addition, r-AtHSP70 protects heart and liver against LPS-induced sepsis preventing hemodynamic portal and cardiac function. Taken together, these data indicate r-AtHSP70 as a natural molecule with protective action against cardiac damage and heart and liver dysfunctions during inflammatory. This may pave the way to clarify the potential clinical application of the novel herbaceous product.
